# Proenkephalin a 119–159 for evaluating glomerular filtration rate and hemodialysis adequacy in patients with end-stage kidney disease: a prospective cohort study

**DOI:** 10.3389/fmed.2026.1762412

**Published:** 2026-04-14

**Authors:** Luobei Zhang, Lin Gong, Chang Hu, Jing Ma, Yanting Zhang, Shixian Zhang, Shuai Hou, Xiang Tong, Zhiyong Peng, Chang Liu

**Affiliations:** 1 Department of Critical Care Medicine, Zhongnan Hospital of Wuhan University, Wuhan, Hubei, China; 2 Hubei Clinical Research Center for Critical Care Medicine, Wuhan, Hubei, China; 3 Department of Critical Care Medicine, Jiayu Hospital Zhongnan Hospital of Wuhan University, Xianning, Hubei, China; 4 School of Basic Medical Sciences, Wuhan University, Wuhan, Hubei, China; 5 Division of Nephrology and Endocrinology, Department of Medicine, Zhongnan Hospital of Wuhan University, Xianning, Hubei, China

**Keywords:** dialysis adequacy, end-stage kidney disease, glomerular filtration rate, hemodialysis, Proenkephalin a 119–159

## Abstract

**Background:**

High levels of Proenkephalin A 119–159 (PENK) have been associated with decreased glomerular filtration rate (GFR) in subjects with preserved kidney function, yet its clinical utility in patients with end-stage kidney disease (ESKD) remains to be established. This study aimed to investigate the correlation between PENK and GFR, and evaluate the clearance of PENK during hemodialysis (HD) in ESKD patients.

**Methods:**

This single-center prospective cohort study enrolled adult patients with ESKD undergoing maintenance HD. Plasma PENK levels were determined before (baseline) and after a single HD session using a double-antibody sandwich enzyme-linked immunosorbent assay. The associations of PENK with estimated GFR (eGFR), and other markers of kidney function and heart failure were analyzed at baseline. Furthermore, changes in PENK levels during HD were evaluated to assess its dialytic clearance.

**Results:**

A total of 100 ESKD patients were enrolled. At baseline, the median eGFR and PENK level were 6.30 mL/min/1.73 m^2^ and 4.88 ng/mL, respectively. In the entire cohort (*n* = 100), no significant correlations were observed between PENK and eGFR (*r* = −0.00, *p* = 0.99), BUN (*r* = 0.16, *p* = 0.10), SCr (*r* = 0.18, *p* = 0.08), NT-proBNP (*r* = −0.06, *p* = 0.58), or LVEF (*r* = 0.12, *p* = 0.26). Among patients with residual urine output (*n* = 56), PENK and eGFR also exhibited no significant correlation (*r* = −0.15, *p* = 0.28). Although significant clearance was observed for BUN (12.59 vs. 3.68 mmol/L, *p* < 0.001) and SCr (458.70 vs. 165.85 μmol/L, *p* < 0.001), PENK levels remained unchanged during HD (4.88 vs. 4.80 ng/mL, *p* = 0.85), with the stability unaffected by residual urine output, dialysis vintage, dialysis frequency, or comorbidities.

**Conclusion:**

In this cohort of ESKD patients, plasma PENK levels showed no significant correlation with eGFR, and negligible clearance of PENK was observed during HD. These preliminary results suggested limited value of PENK for evaluating residual kidney function and dialysis adequacy in patients with ESKD. Further studies are required to clarify the mechanisms by which PENK is involved in kidney impairment and to characterize PENK kinetics during HD in this patient population.

## 1 Introduction

Chronic kidney disease (CKD) represents a substantial global health burden, according to 2023 estimates, the age-standardized prevalence of CKD in adults is 14.2% worldwide, while end-stage kidney disease (ESKD), its most severe form, affects approximately 0.2% of the global population (1). Most ESKD patients rely on maintenance hemodialysis (HD) for survival. Accumulating evidence has demonstrated that residual kidney function (RKF) and dialysis adequacy (DA) are closely associated with survival and quality of life (2–6). However, routine RKF measurement remains clinically limited, primarily due to the inconvenience of urine collection required for clearance estimation (7). Meanwhile, conventional DA markers [e.g., serum creatinine (SCr) and blood urea nitrogen (BUN)] exhibit poor specificity and sensitivity, as their levels are confounded by hydration status, muscle mass, metabolic rate, malnutrition, and medication use (5, 8, 9). Thus, identifying convenient and reliable biomarkers to optimize RKF and DA assessment is urgently needed.

Proenkephalin A 119–159 (PENK) is a stable enkephalin surrogate which has emerged as a novel biomarker of kidney function and injury (10). With a low molecular weight of 4.5 kDa, PENK is freely filtered by the glomerulus with minimal tubular interference, and its plasma stability is unaffected by age, sex, or protein binding (11, 12), making it an attractive candidate for evaluating glomerular function (12). Large cohort studies have reported strong correlations between plasma PENK concentrations and measured glomerular filtration rate (GFR) in patients with stable and declining kidney function (13, 14). Elevated baseline PENK levels in healthy individuals have also been shown to predict long term estimated GFR (eGFR) decline and incident CKD (15, 16).

Although the association between PENK and glomerular dysfunction has been established in non- ESKD patients, its relationship with GFR in patients with ESKD remains poorly defined. Furthermore, *ex vivo* studies have suggested PENK can be effectively cleared during renal replacement therapy (RRT) (17), but Grycuk et al. reported paradoxically higher PENK levels post-dialysis than pre-dialysis in ESKD patients, implying limited dialyzability of PENK (18). These conflicting findings underscore the need to clarify whether PENK is cleared during HD and to explore its utility as a marker of RKF or DA in the ESKD population.

To address these knowledge gaps, this prospective observational study was conducted in ESKD patients with the following aims: (1) to evaluate the clearance of PENK during HD, and (2) to investigate the correlation between plasma PENK levels and eGFR, as well as other kidney function parameters.

## 2 Methods

### 2.1 Study design and setting

This single-center, prospective observational study was conducted at the Dialysis Unit of Zhongnan Hospital of Wuhan University, Jiayu Branch (Hubei, China). The unit operating 36 stations, serves 100–150 maintenance HD patients annually. The Institutional Research Ethics Committee approved the study protocol (No. 20250001); written informed consent was obtained from all participants prior to enrollment.

### 2.2 Participants

We screened all maintenance HD recipients in our unit over a 4-month period (March 1 to June 30, 2025). The inclusion criteria were: (1) age ≥18 years, (2) diagnosed with ESKD, (3) receiving HD treatments ≥2 times weekly for >90 days prior to screening, and (4) willing and able to provide written informed consent. The exclusion criteria involved: (1) history of organ transplantation, (2) vulnerable adults, prisoners, or had known pregnancies, (3) received other RRT during the study period, (4) acute exacerbation of kidney disease or acute intercurrent illness, (5) inability to complete the HD session, (6) inability to provide written informed consent, and (7) incomplete or missing clinical data.

### 2.3 Data collection

We collected demographic and clinical data (diagnosis, comorbidities, dialysis vintage and frequency) from the electronic health records. Before HD, the height (cm), weight (kg), and blood pressure (mmHg) of the patients were measured, and questions concerning socio-economic status, lifestyle factors, health status, medical history, and residual urine volume were answered by the patients or their healthcare proxy via an interviewer-administered questionnaire by a trained nurse. Following a 10-min rest period, transthoracic echocardiogram was performed, and left ventricular ejection fraction (LVEF) was assessed using standard formulas by an experienced ultrasound physician. Blood samples were collected immediately before and at the end of the HD session, each sample was divided into two aliquots, one part was transferred to the hospital’s central laboratory for the measurement of N-terminal pro b-type natriuretic peptide (NT-proBNP), BUN, and SCr, the other part was immediately centrifuged at 3000 rpm for 10 min at 4 °C, the obtained plasma was transferred into 1.5 mL microcentrifuge tubes and stored at −20 °C until the assay of PENK. Plasma NT-proBNP was measured only before HD, whereas BUN, SCr, and PENK were measured both before and after HD. Patients with <100 mL/day urine output (UO) were regarded as having zero GFR, for patients with residual UO (≥100 mL/d), eGFR was calculated using the 2009 Chronic Kidney Disease Epidemiology Collaboration (CKD-EPI) equation (19), where SCr value was the mean of all pre-dialysis SCr measurements within 90 to 7 days before the index HD, if no measurement was available within this window, the immediately before HD SCr value was employed. Heart failure (HF) diagnosis and classification conformed to contemporary guideline-directed standards (20).

### 2.4 HD protocol

HD was performed according to current practice guidelines (21, 22) by a team of nephrologist, registered nurses, and technicians. HD was delivered via Dialog+ platform (B. Braun Medical. Bethlehem, United States) using REXEED 18-UC filter (Asahi Kasei Medical, Hangzhou, China). The settings were uniform for all patients, with adjustments only for clinical emergencies, the key parameters included: (1) blood flow rate: 200–400 mL/min, (2) dialysate flow rate: 500–800 mL/min, (3) treatment duration: 4–6 h per session, (4) anticoagulation: intravenous unfractionated heparin with initial bolus 20 IU/kg, and maintenance infusion 5–15 IU/kg/h, or regional citrate anticoagulation, as appropriate, (5) ultrafiltration rate: 6–12 mL/kg/h, adjusted according to the patient’s fluid retention status and capped at 13 mL/kg/h, (6) dialysate composition: Na^+^ 137 mmol/L, K^+^ 2.0 mmol/L, Ca^2+^ 1.5 mmol/L, Mg^2+^ 0.5 mmol/L, Cl^−^ 108 mmol/L, HCO_3_^−^31 mmol/L, and CH3COO^−^ 4.0 mmol/L.

### 2.5 Measurement of PENK

The measurements were performed in batch within 1 months after recruitment of the last patient by technicians blinded to clinical data. PENK was measured in duplicates using a double-antibody sandwich enzyme-linked immunosorbent assay (ELISA) employing monoclonal antibodies specific to human PENK peptide (PENK ELISA Kit, Xinweiyu Biotechnology, Shanghai, China). The principles and methodology of this assay have been previously described in detail (11, 18). The lower detection limit of the assay was 0.1 ng/mL. The intra-assay and inter-assay coefficients of variation were 7.1% (10 replicates) and 9.5% (15 runs over 3 days), respectively.

### 2.6 Statistical analysis

Categorical variables were presented as counts (*n*) and percentages (%), continuous variables were expressed as medians with interquartile ranges (IQR) or means with standard deviations (SD), as appropriate. For the pre- and post-HD comparison, the Wilcoxon signed-rank test was used for non-normally distributed data. Comparisons between the two or more groups were conducted by the Wilcoxon rank-sum test or Kruskal-Wallis test, pairwise comparisons were performed using Dunn’s test with Bonferroni correction. The correlation between two variables was examined by the Pearson correlation or Spearman’s rank correlation, as appropriate. A two-tailed *p*-value < 0.05 was considered statistically significant. All statistical analyses were performed using SPSS version 29 (IBM Corp., Armonk, United States).

## 3 Results

### 3.1 Baseline characteristics

**Figure 1:**
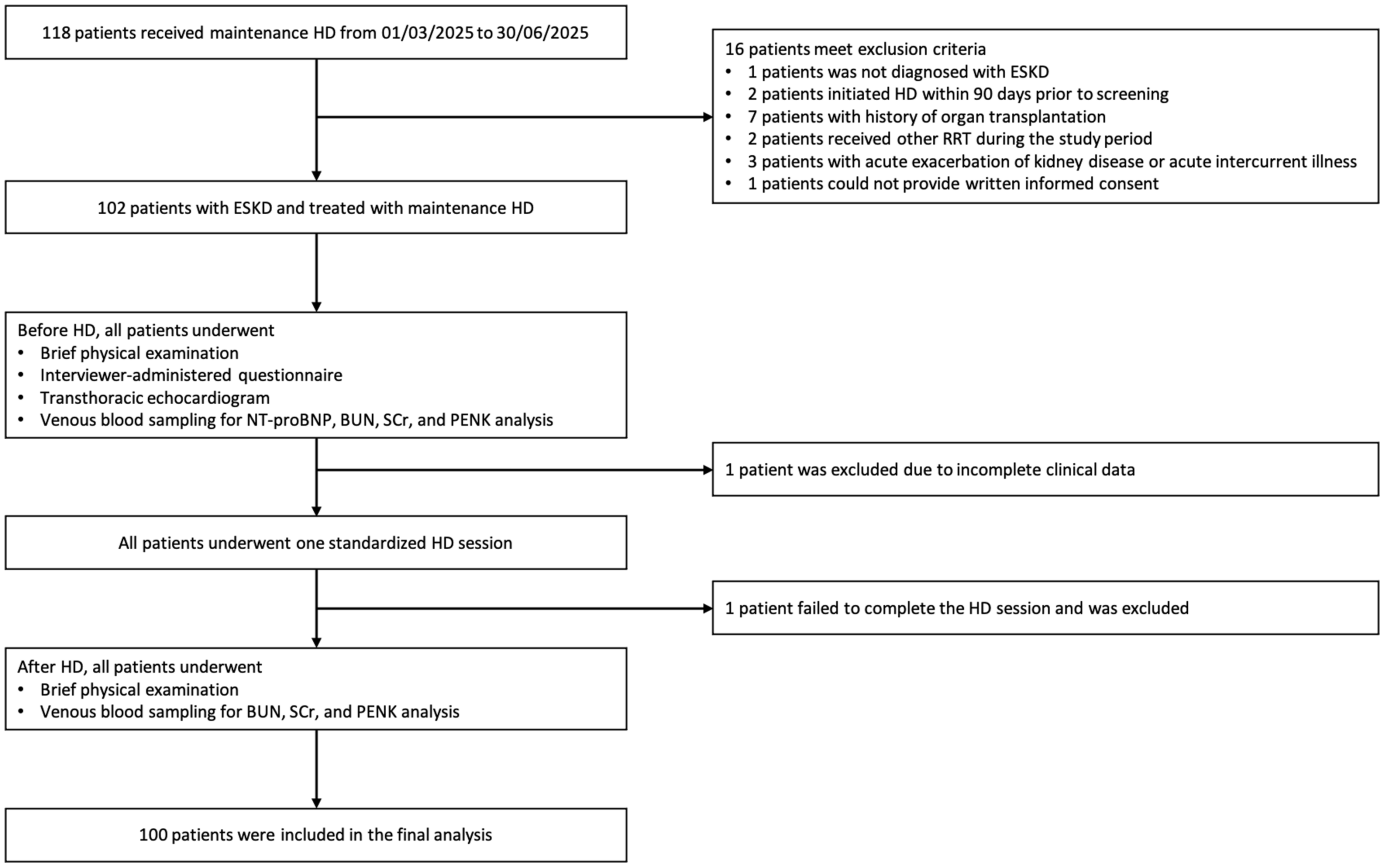
Participant selection and inclusion flowchart. HD, hemodialysis; ESKD, end-stage kidney disease; RRT, renal replacement therapy; NT-proBNP, N-terminal pro B-type natriuretic peptide; BUN, blood urea nitrogen; SCr, serum creatinine; PENK, proenkephalin A 119–159.

After screening, a total of 100 patients comprised the analytical cohort (Figure 1). As summarized in Table 1, the mean age of the cohort was approximately 59 years, with the majority being male (65%). Majority of the patients (80%) had no or very low residual UO (< 500 mL/d) and received HD thrice weekly (69%). The distribution of the dialysis vintage was as follows, <1 year (27%), 1–5 years (38%), 5–10 years (23%), and >10 years (12%). Hypertension (HTN, 78%) and HF (31%) represented the most common comorbidities, despite the pre-dialysis median NT-proBNP level raised to 3638.50 pg./mL, most patients (94%) demonstrated preserved LVEF in echocardiogram. The median eGFR and pre-HD PENK levels of the cohort were 6.30 mL/min/1.73 m^2^ and 4.88 ng/mL, respectively, for patients with residual UO (*n* = 56), the median eGFR and pre-HD PENK levels were 10.50 mL/min/1.73 m^2^ and 4.96 ng/mL, respectively.

**Table 1:** Demographics and baseline characteristics of the studied cohort.

Variables	*N*	Values
Male sex	100	65 (65)
Age, years	100	59.17 ± 12.67
BMI, kg/m^2^	100	22.12 ± 3.36
Dialysis vintage, years	100	
<1 year		27 (27)
≥1 and <5 years		38 (38)
≥5 and <10 years		23 (23)
≥10 years		12 (12)
Dialysis frequency	100	
Twice a week		13 (13)
Thrice a week		69 (69)
Five times every 2 weeks		18 (18)
Residual urine output	100	
<100 mL/d		44 (44)
≥100 and <500 mL/d		36 (36)
≥500 and <1,000 mL/d		20 (20)
eGFR, mL/min/1.73 m^2^		
Entire cohort	100	6.30 (0.00, 11.50)
Patients with residual urine output	56	10.50 (7.90, 17.40)
SCr, umol/L	100	458.70 (346.58, 585.85)
BUN, mmol/L	100	13.02 ± 5.31
PENK, ng/mL		
Entire cohort	100	4.88 (4.25, 5.60)
Patients with residual urine output	56	4.96 (4.21, 6.43)
Comorbidity	100	
Hypertension		78 (78)
Heart failure		31 (31)
Coronary artery disease		16 (16)
Diabetes mellitus		13 (13)
LVEF value, %	99	64.00 (61.25, 68.00)
LVEF category	99	
<40%		3 (3.03)
≥40% and <50%		3 (3.03)
≥50%		93 (93.94)
NT-proBNP, pg./mL	100	3638.50 (1356.25, 13568.50)

Numbers indicate as *n* (%), median (IQR), or mean ± SD, where applicable. BMI, body mass index; eGFR, estimated glomerular filtration rate; SCr, serum creatinine; BUN, blood urea nitrogen; PENK, proenkephalin A 119–159; LVEF, left ventricular ejection fraction, NT-proBNP, N-terminal pro b-type natriuretic peptide.

### 3.2 Correlations of PENK with demographic characteristics, comorbidities, and markers of cardiac and kidney function

**Figure 2:**
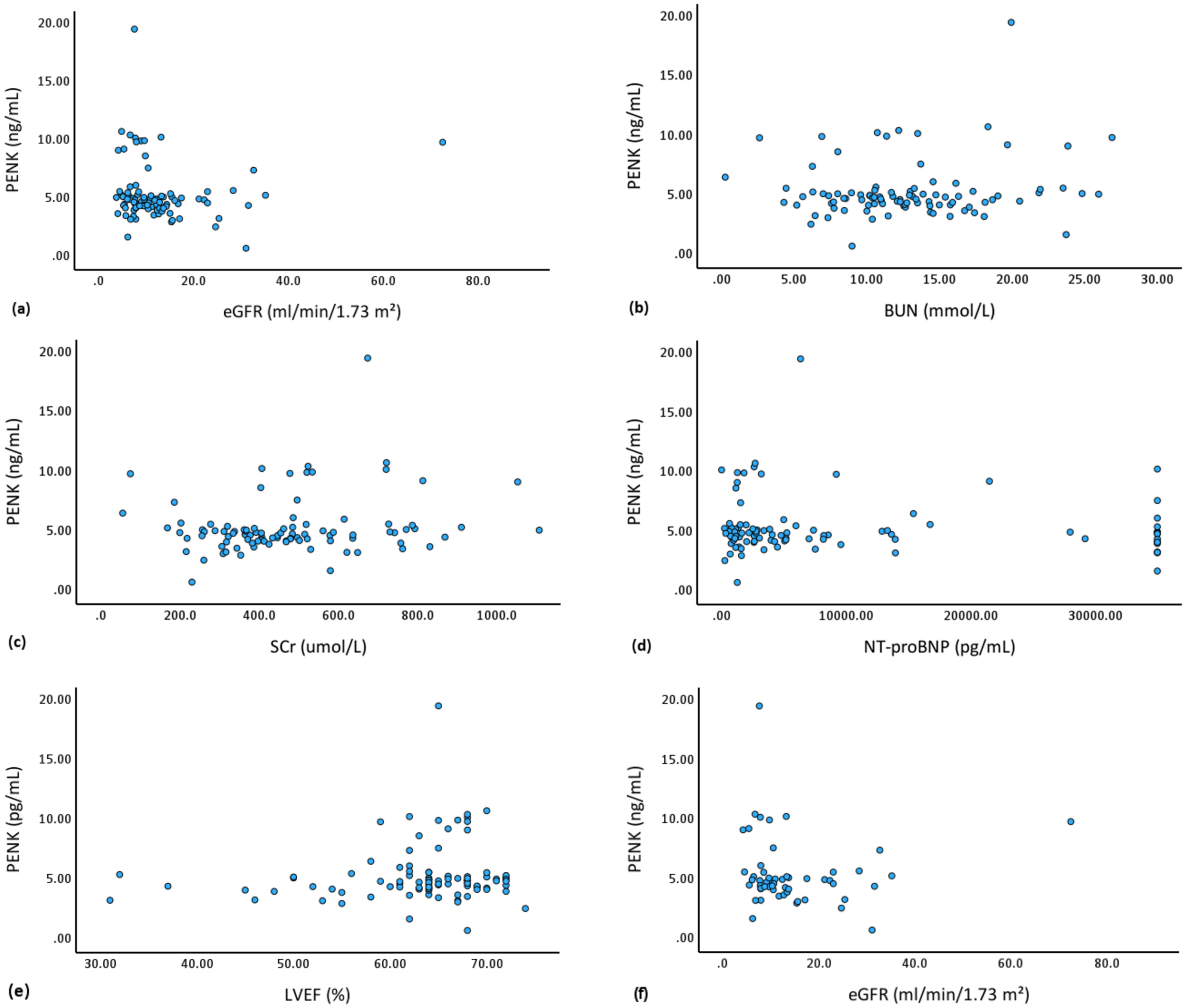
Scatter plots demonstrate the relationship between pre-HD PENK levels and **(a)** eGFR of the entire cohort (*r* = −0.00, *p* = 0.99), **(b)** BUN (*r* = 0.16, *p* = 0.10), **(c)** SCr (*r* = 0.18, *p* = 0.08), **(d)** NT-proBNP (*r* = −0.06, *p* = 0.58), **(e)** LVEF (*r* = 0.12, *p* = 0.26), and **(f)** eGFR of the patients with residual urine output (*r* = −0.15, *p* = 0.28). HD, hemodialysis; PENK, proenkephalin A 119–159; eGFR, estimated glomerular filtration rate; BUN, blood urea nitrogen; SCr, serum creatinine; NT-proBNP, N-terminal pro B-type natriuretic peptide; LVEF, left ventricular ejection fraction.

**Figure 3:**
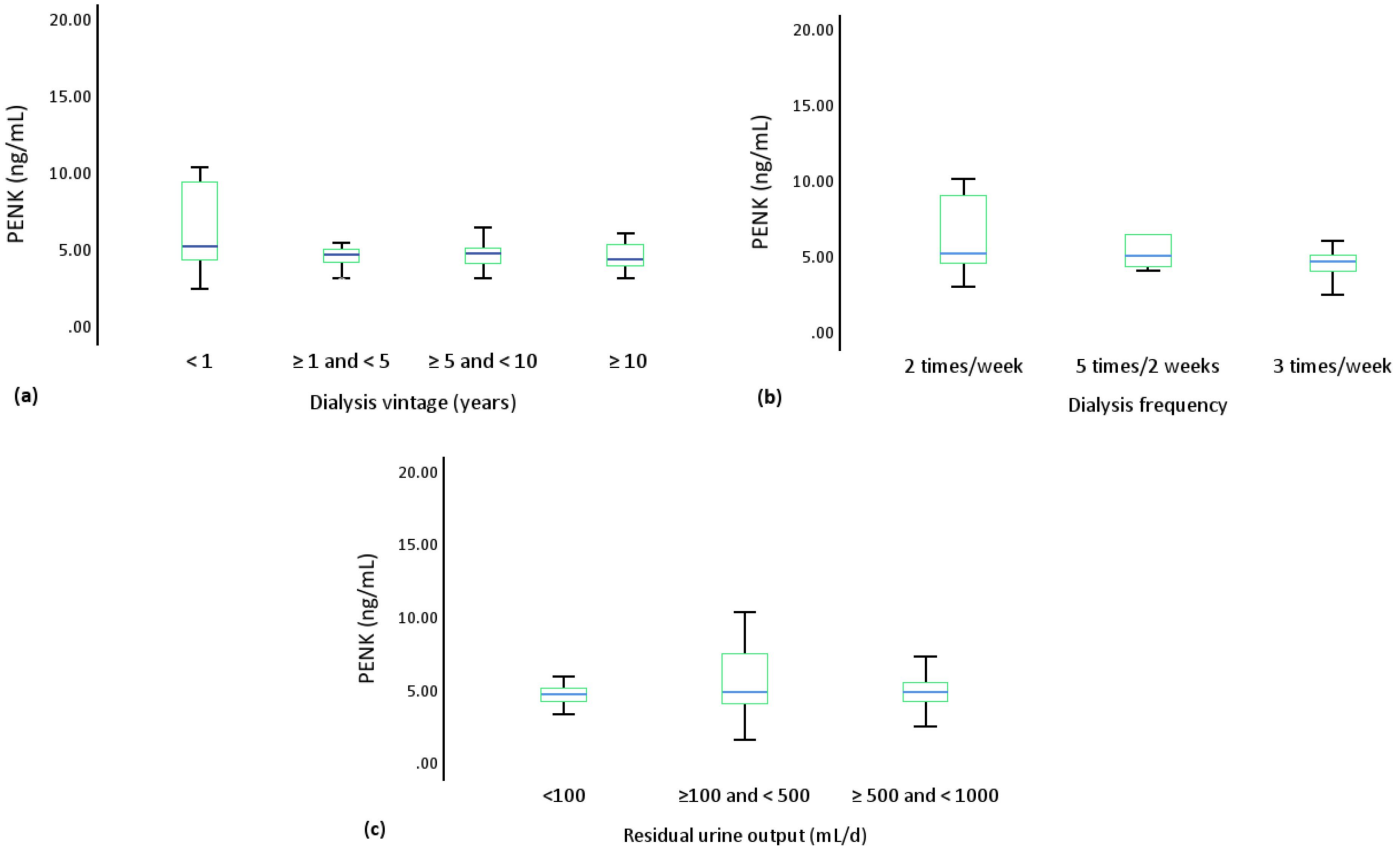
Pre-HD PENK levels were analyzed according to **(a)** dialysis vintage (4.27 ng/mL vs. 4.80 ng/mL vs. 4.86 ng/mL vs. 4.46 ng/mL, *p* = 0.27), **(b)** dialysis frequency (5.29 ng/mL vs. 5.13 ng/mL vs. 4.75 ng/mL, *p* = 0.06), and **(c)** residual urine output (4.79 ng/mL vs. 4.96 ng/mL vs. 4.96 ng/mL, *p* = 0.85). HD, hemodialysis; PENK, proenkephalin A 119–159.

Before HD, the PENK levels were similar in male and female patients (4.75 ng/mL vs. 4.99 ng/mL, *p* = 0.47), and showed no significant correlations with age (*r* = − 0.03, *p* = 0.75) and body mass index (*r* = 0.02, *p* = 0.87) (Supplementary Figure 1). The PENK levels were unaffected by the presence of HTN (*p* = 0.81), HF (*p* = 0.45), coronary artery disease (*p* = 0.61), and diabetes mellitus (*p* = 0.92) (Supplementary Figure 2). As illustrated in Figure 2, no significant correlations were observed in the entire cohort between PENK and eGFR (*r* = −0.00, *p* = 0.99), BUN (*r* = 0.16, *p* = 0.10), SCr (*r* = 0.18, *p* = 0.08), NT-proBNP (*r* = −0.06, *p* = 0.58), or LVEF (*r* = 0.12, *p* = 0.26). Among patients with residual UO, PENK and eGFR also showed no significant correlation (*r* = −0.15, *p* = 0.28). The distribution of PENK levels were similar in patients with different dialysis vintage (*p* = 0.12), dialysis frequency (*p* = 0.08), and residual UO (*p* = 0.67) (Figure 3).

### 3.3 The change of PENK levels before and after HD

The body weight (59.55 kg vs. 57.00 kg, *p* < 0.001), BUN (12.59 mmol/L vs. 3.68 mmol/L, *p* < 0.001), and SCr (458.70 μmol/L vs. 165.85 μmol/L, *p* < 0.001) of the patients decreased significantly after one HD session. However, the PENK levels were similar before and after HD (4.88 ng/mL vs. 4.80 ng/mL, *p* = 0.85) (Table 2). Further analyses confirmed consistent pre- to post-HD changes in PENK levels across all subgroups. No significant differences were observed based on gender, residual UO, dialysis vintage, dialysis frequency, or LVEF. Furthermore, the presence of HTN, HF, coronary artery disease, or diabetes mellitus did not significantly affect these changes (Table 3).

**Table 2:** Changes in body weight, BUN, SCr, and PENK after a single HD session.

Variables	Before HD (*n* = 100)	After HD (*n* = 100)	*p* ^†^
Body weight, kg	59.55 (52.90, 64.48)	57.00 (50.70, 62.65)	<0.001
BUN, mmol/L	12.59 (9.59, 16.11)	3.68 (0.79, 5.27)	<0.001
SCr, μmol/L	458.70 (346.58, 585.85)	165.85 (87.23, 238.43)	<0.001
PENK, ng/ml	4.88 (4.25, 5.60)	4.80 (4.21, 5.48)	0.85

Numbers indicate as median (IQR). HD, hemodialysis; BUN, blood urea nitrogen; SCr, serum creatinine; PENK, proenkephalin A 119–159. ^†^*p*-values come from Wilcoxon signed-rank test.

**Table 3:** Changes in PENK levels following a single HD session across subgroups.

Variables	*N*	Pre-HD PENK (ng/mL)	Post-HD PENK (ng/mL)	*p* ^†^
Gender				
Male	65	4.75 (4.24, 5.55)	4.83 (4.25, 5.57)	0.27
Female	35	4.99 (4.23, 5.61)	4.54 (4.07,5.33)	0.23
Residual urine output				
<100 mL/d	44	4.84 (4.28, 4.24)	4.52 (4.07, 5.01)	0.26
≥100 and <500 mL/d	36	4.95 (4.19, 8.39)	4.91 (4.24, 8.85)	0.28
≥500 and <1,000 mL/d	20	4.95 (4.32, 5.67)	5.00 (4.29, 5.49)	0.79
Dialysis vintage				
<1 year	27	5.27 (4.47, 9.84)	5.23 (4.47, 9.47)	0.79
≥1 and <5 years	38	4.80 (4.28, 5.16)	4.83 (4.11, 5.44)	0.60
≥5 and <10 years	23	4.86 (4.17, 5.22)	4.65 (3.90, 5.07)	0.81
≥10 years	12	4.46 (4.04, 5.54)	4.48 (4.22, 4.79)	0.81
Dialysis frequency				
Twice a week	13	5.29 (4.63, 9.20)	5.32 (4.54, 9.08)	0.70
Five times every 2 weeks	18	5.13 (4.44, 7.36)	5.27 (4.43, 8.06)	0.40
Thrice a week	69	4.75 (4.11, 5.23)	4.71 (4.05, 5.25)	0.94
Hypertension				
Yes	78	4.85 (4.23, 6.05)	4.80 (4.16, 5.53)	0.37
No	22	4.93 (4.27, 5.52)	4.67 (4.22, 5.16)	0.15
Heart failure				
Yes	31	4.84 (4.20, 5.42)	4.91 (4.25, 5.50)	0.13
No	69	4.89 (4.26, 5.65)	4.79 (4.09, 5.44)	0.30
LVEF				
<50%	6	4.07 (3.30, 4.69)	4.39 (4.00, 4.81)	0.46
≥50%	93	4.90 (4.32, 5.63)	4.82 (4.20, 5.53)	0.97
Coronary artery disease				
Yes	16	4.90 (4.19, 5.20)	5.14 (3.75, 5.59)	0.64
No	84	4.88 (4.25, 5.68)	4.78 (4.21, 5.46)	0.99
Diabetes mellitus				
Yes	13	4.89 (4.43, 5.47)	4.71 (3.77, 5.41)	0.55
No	87	4.87 (4.20, 5.60)	4.80 (4.21, 5.50)	0.70

Numbers indicate as median (IQR). HD, hemodialysis; PENK, proenkephalin A 119–159; LVEF, left ventricular ejection fraction. ^†^*p*-values come from Wilcoxon signed-rank test.

## 4 Discussion

In this prospective observational analysis of ESKD patients undergoing HD, plasma PENK levels were consistently elevated above the normal range. However, no significant correlations were observed between PENK and eGFR, or other markers of cardiac and kidney function. PENK demonstrated trans-HD stability, and unaffected by residual UO, dialysis vintage, dialysis frequency, or comorbidities.

Previous studies have demonstrated a strong correlation between PENK and measured GFR in patients with steady or impaired kidney function (23, 24), as well as elevated PENK levels in patients with CKD relative to those with preserved kidney function (11, 25), but the PENK-eGFR correlation remains poorly characterized in the ESKD population, as these patients have been systematically excluded from prior investigations (23). To the best of our knowledge, only Grycuk et al. and we have specifically examined PENK as a kidney biomarker in ESKD patients, with neither identifying a significant PENK-eGFR correlation (18). It has been reported that PENK levels may vary according to certain patient characteristics and comorbidities, particularly elevated in patients with older age, HTN or HF (26, 27). However, in the present study, we observed no significant correlations between PENK levels and demographic characteristics, comorbidities, LVEF, or NT-proBNP levels. Although PENK has been associated with HF severity and adverse outcomes in previous investigations (28, 29), large-scale trials adjusted for kidney function revealed PENK lacked independent predictive value for new-onset HF in the general population (25), as well as for mortality or rehospitalization in patients with acute or chronic HF (30). Collectively, these findings suggest that in ESKD patients, among whom HTN, diabetes, and HF are highly prevalent, PENK not only lacks reliability as a GFR marker but also appears limited utility for cardiac risk stratification or prognostication.

PENK was presumed to be freely removed by RRT and proposed as a biomarker for DA (10). However, neither the investigation by Grycuk et al. nor our study observed a significant post-HD reduction in PENK levels among ESKD patients. In contrast, Lorenzin et al. demonstrated effective clearance of PENK via continuous RRT in an *ex vivo* simulation, reporting a sieving coefficient of 1.04 and a diffusive clearance rate of 23.08 (17), which were similar to those observed for BUN and SCr (31). Notably, divergent filters were used in these studies, Lorenzin et al. employed the Ultraflux AV1000S (Fresenius Medical Care, Bad Homburg, Germany) (17), while we used the REXEED 18-UC, both membranes demonstrate efficient sieving for solutes ≤30 kDa (17, 32). Grycuk et al. reported elevated post-HD PENK levels in patients receiving both high- and low-flux HD, with significantly higher PENK levels were documented in the low-flux subgroup (18). This finding likely reflected reduced clearance of PENK during low-flux HD, consistent with its characterization (4.5 kDa) as a medium-molecular-weight solute. Thus, variability in RRT modality and membrane permeability likely contributed to the discrepancies in results. Furthermore, unlike the *ex vivo* simulation by Lorenzin et al., in which the net fluid balance was maintained at zero (18), clinical HD necessitates net ultrafiltration. Therefore, a plausible hypothesis is that in both the studies by Grycuk et al. and us, the fluid loss potentially exceeded the PENK clearance. This may partly account for the observed stability or even paradoxical increase in PENK levels following HD.

Besides PENK, Plasma proteins such as cystatin C, *β*-2 microglobulin, and β-trace protein have been proposed as potential candidate markers for RKF or DA (7, 33). Consistent with our findings, previous investigations had reported that the correlations between these plasma markers and GFR were significantly weakened at low GFR levels, particularly below 5 mL/min (7, 34). These results indicated that current candidate biomarkers had limited indicative value for RKF in patients with severe kidney impairment. Further research is therefore warranted to improve the performance of these biomarkers or to identify novel biomarkers that can better distinguish varying degrees of kidney impairment.

Of particular interest, PENK was previously considered biologically inactive with minimal tubular involvement (35), but recent evidence challenges this view. PENK expression has been identified in rat renal tubulars *ex vivo* (36), and, more recently, Liu et al. demonstrated its expression in zebrafish renal proximal tubular epithelial cells, and indicated a role of PENK in regulating kidney regeneration via hydrogen peroxide production control (37). These findings highlight the bioactivity of PENK and its potential regulatory role in normal kidney function or kidney diseases (36–39). The precise metabolic kinetics and functional role of PENK in humans remain unclear. Future studies are warranted to clarify whether PENK plays a role in modulating kidney function across different stages of kidney disease and to characterize the kinetic profiles of PENK during HD in patients with ESKD.

Our study has several limitations. First, the single-center observational design and relatively small sample size limited statistical power and increase vulnerability to unmeasured confounders and residual bias. Second, we were unable to assess correlations between PENK and directly measured GFR; reliance on SCr–based eGFR may inadequately reflect RKF in ESKD patients. Third, although a standardized HD protocol was followed, variations in delivered dialysis dose and concurrent clinical interventions could not be fully controlled, which might confound associations. Fourth, despite markedly elevated plasma NT-proBNP levels, preserved LVEF was observed in 94% of participants, consistent with previous evidence implicating volume overload as the primary driver of NT-proBNP elevation in this population rather than impaired cardiac contractility (40). This low prevalence of advanced cardiac dysfunction limited our ability to meaningful analysis of PENK’s relationship with severe cardiac impairment. Finally, PENK is eliminated renally, varying levels of residual UO within the cohort may confound plasma PENK concentrations, potentially introducing bias.

## 5 Conclusion

In this cohort of patients with ESKD, we failed to demonstrate significant correlations between PENK and eGFR or other established kidney function markers, nor did we observe significant reductions in circulating PENK levels following HD. Collectively, these data suggest limited value of PENK in assessing RKF or DA in patients with ESKD. Given the inherent pathophysiological heterogeneity of ESKD and the observational nature of the present study, our findings should be interpreted as preliminary and hypothesis-generating. Further research is required to elucidate PENK kinetics and untangle its association with kidney function in this specific patient population.

## Data Availability

The original data supporting the findings are openly available in Figshare with DOI: 10.6084/m9.figshare.31925520 and direct access URL: https://doi.org/10.6084/m9.figshare.31925520. The shared data only include ELISA experimental data (no patient privacy or sensitive information), ensuring research transparency and reproducibility.

## Glossary

BUN: Blood urea nitrogen
CKD: Chronic kidney disease
CKD-EPI: Chronic Kidney Disease Epidemiology Collaboration
DA: Dialysis adequacy
eGFR: Estimated glomerular filtration rate
ESKD: End-stage kidney disease
ELISA: Enzyme-linked immunosorbent assay
GFR: Glomerular filtration rate
HD: Hemodialysis
HF: Heart failure
HTN: Hypertension
IQR: Interquartile ranges
LVEF: Left ventricular ejection fraction
NT-proBNP: N-terminal pro b-type natriuretic peptide
UO: Urine output
PENK: Proenkephalin A 119–159
RKF: Residual kidney function
RRT: Renal replacement therapy
SCr: Serum creatinine
SD: standard deviations
