# Supplementary material for: Proenkephalin a 119–159 for evaluating glomerular filtration rate and hemodialysis adequacy in patients with end-stage kidney disease: a prospective cohort study
Source: Front Med (Lausanne). 2026 Apr 14;13:1762412. doi: 10.3389/fmed.2026.1762412 (PMC13120930; doi:10.3389/fmed.2026.1762412)
Supplement: Supplementary file 1 [file Data_Sheet_1.DOCX]

**Supplemental Digital Content**

**Proenkephalin A 119-159 for Evaluating Glomerular Filtration Rate and Hemodialysis Adequacy in Patients with End-Stage Kidney Disease: A** **Prospective Cohort Study**

Luobei Zhang, Lin Gong, Chang Hu, Jing Ma, Yanting Zhang, Shixian Zhang, Shuai Hou, Xiang Tong, Zhiyong Peng, Chang Liu

Corresponding author:

Chang Liu, MD, PhD
Department of Critical Care Medicine

Zhongnan Hospital of Wuhan University

Wuhan, Hubei, 430071, China
E-mail: changliuwhu@whu.edu.cn


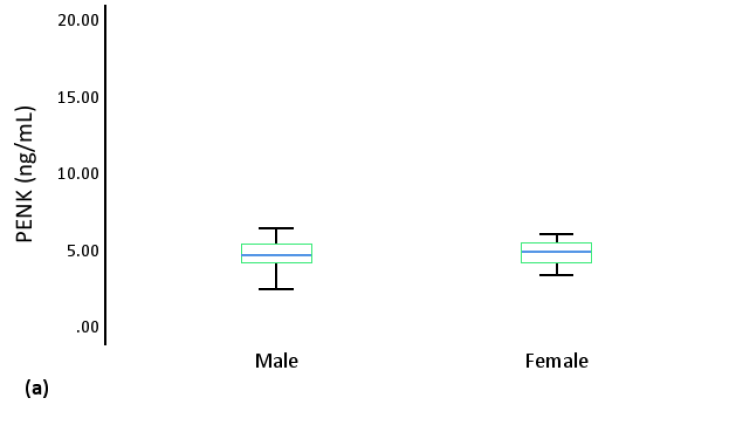


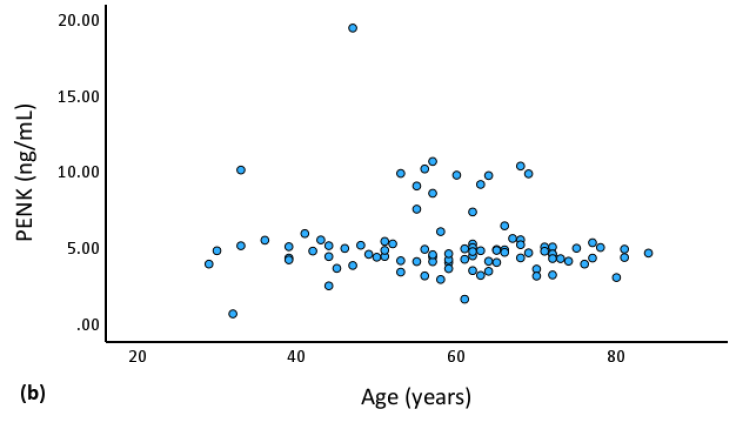


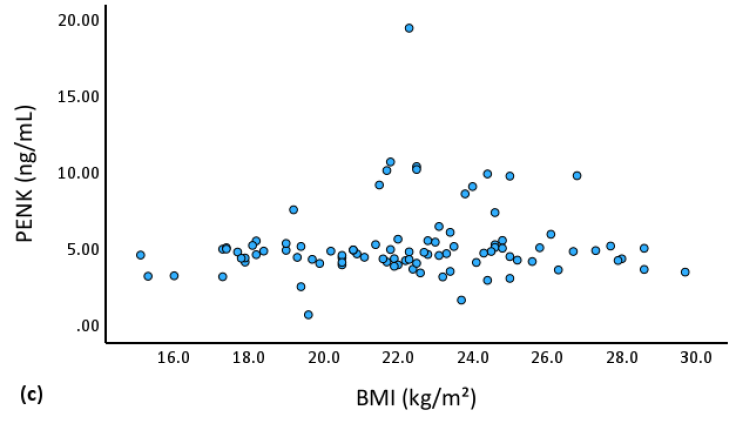


**Supplemental Figure 1.** (a) Comparison of pre-HD PENK levels between male and female patients (4.75 ng/mL vs. 4.99 ng/mL, *P* = 0.47). (b) Correlation between pre-HD PENK levels and age (*r* = -0.03, *P* = 0.75). (c) Correlation between pre-HD PENK levels and BMI (*r* = 0.02, *P* = 0.87). Abbreviations: HD, hemodialysis; PENK, proenkephalin A 119-159; BMI, Body Mass Index.


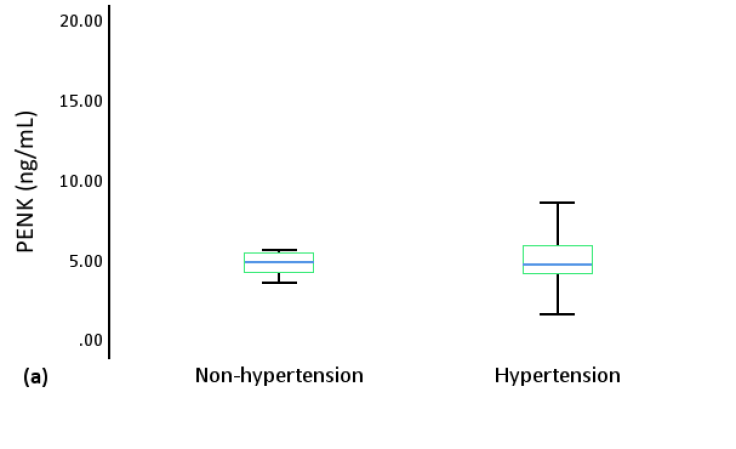

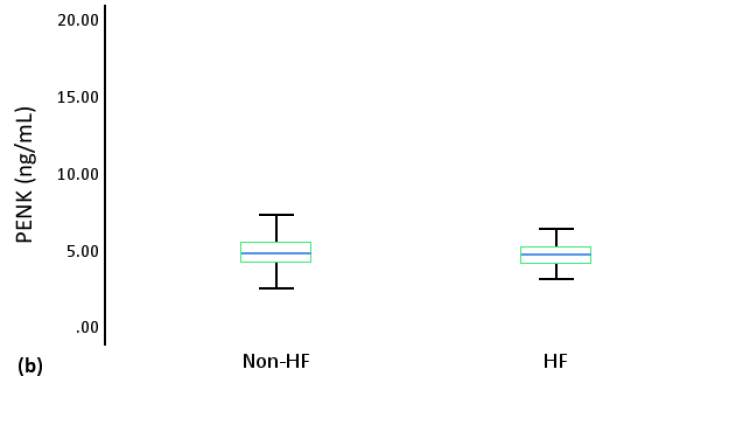

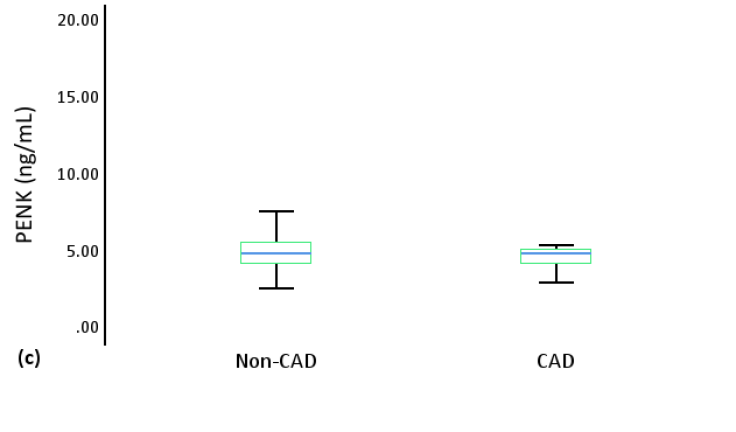

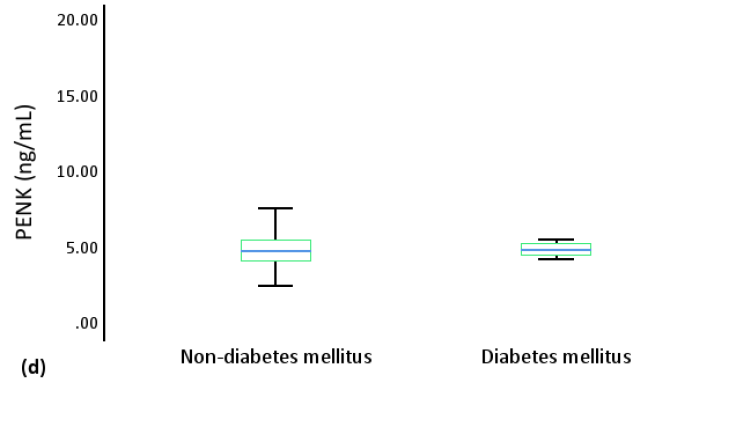


**Supplemental Figure 2.** Pre-HD PENK levels were compared between patients with and without specific comorbidities: (a) hypertension (4.93 ng/mL vs. 4.85 ng/mL, *P* = 0.81), (b) HF (4.89 ng/mL vs. 4.84 ng/mL, *P* = 0.45), (c) coronary artery disease (4.88 ng/mL vs. 4.90 ng/mL, *P* = 0.61), and (d) diabetes mellitus (4.87 ng/mL vs. 4.89 ng/mL, *P* = 0.92). Abbreviations: HD, hemodialysis; PENK, proenkephalin A 119-159; HF, heart failure; CAD, coronary artery disease.
